# The Economic Value of Rotavirus Vaccination When Optimally Implemented in a High-Income Country

**DOI:** 10.3390/vaccines11050917

**Published:** 2023-04-28

**Authors:** Baudouin Standaert

**Affiliations:** 1 Department Care and Ethics, Faculty of Medicine and Life Sciences, University Hasselt, 3590 Diepenbeek, Belgium; baudouin.standaert@skynet.be; 2 HEBO bv, 2020 Antwerpen, Belgium

**Keywords:** rotavirus vaccination, economic evaluation long-term, optimal introduction cost-impact analysis

## Abstract

Rotavirus vaccination was introduced in high-income countries starting in 2006, with no recommendation for optimal implementation. Economic evaluations were presented before launch projecting potential impacts. Few economic reassessments have been reported following reimbursement. This study compares the short- to long-term economic value of rotavirus vaccination between pre-launch predictions and real-world evidence collected over 15 years, proposing recommendations for optimal vaccine launch. A cost-impact analysis compared rotavirus hospitalisation data after the introduction of vaccination between pre-launch modelled projections and observed data collected in the RotaBIS study in Belgium. A best model fit of the observed data was used to simulate launch scenarios to identify the optimal strategy. Data from other countries in Europe were used to confirm the potential optimal launch assessment. The Belgian analysis in the short term (first 8 years) indicated a more favourable impact for the observed data than predicted pre-launch model results. The long-term assessment (15 years) showed bigger economic disparities in favour of the model-predicted scenario. A simulated optimal vaccine launch, initiating the vaccination at least 6 months prior the next seasonal disease peak with an immediate very high vaccine coverage, indicated important additional potential gains, which would make vaccination very cost impactful. Finland and the UK are on such a route leading to long-term vaccination success, whereas Spain and Belgium have difficulties in achieving optimum vaccine benefits. An optimal launch of rotavirus vaccination may generate substantial economic gains over time. For high-income countries that are considering implementing rotavirus vaccination, achieving an optimal launch is a critical factor for long-term economic success.

## 1. Introduction

Economic evaluations of new vaccines coming onto the market are often developed and published prior to authorisation and launch, based on summary efficacy data from randomised controlled clinical trials conducted in places where the vaccine will be first administered [1,2,3]. Such economic assessments present potential value estimates with assumptions made about the long-term vaccine effect [4]. They provide important information with a cost-effectiveness analysis that influences price-setting for the new product at market launch. The evaluations are supported by extended sensitivity analyses of the variables subject to uncertainties. This approach is well established and recommended in guidelines, and the evaluations are applicable in countries that wish to assess the economic value of the new products to reimburse, as local authorities are willing to pay to the vaccine producer a vaccine price worth its economic value [5]. However, it is surprising to observe that these early economic evaluations are rarely challenged by data collected subsequent to the approval and implementation of the new product [6,7]. Moreover, if the initial assessment is simple in its presentation, it is likely that long-term evaluations will not be questioned [8,9].

However, effect monitoring of new vaccines in real-life settings is essential to obtain accurate economic value estimates in the short to long term [10]. Results based on observed data should be compared with projections made at vaccine submission when aiming for reimbursement [11]. This is particularly relevant for preventative vaccinations, as the potential gain could be affected by many different factors impacting the long-term benefit, which are unknown prior to launch. Rotavirus vaccination provides a perfect example of the need for long-lasting monitoring and evaluation.

Different vaccines are available on the market against rotavirus infection, of which two are predominant in high-income countries: a two-dose human assorted live-attenuated vaccine called Rotarix (GSK), and a three-dose live-attenuated human–bovine assorted vaccine called RotaTeq (Merck) [12,13]. It is assumed that the effect over time is equivalent between both vaccines [14]. Before the start of this vaccination programme against rotavirus, it was generally considered that this disease (diarrhoea in children) was easy to manage in high-income countries with a low mortality rate [15,16,17]. The vaccine had a major positive effect on hospitalisations observed in the clinical trial data [1]. Its administration was straightforward because of its oral formulation. However, when observed, real-world vaccine effect data were collected and scrutinised in detail, the actual impacts of the vaccination and the disease were difficult to understand. Real-world data were collected in a special study set up in Belgium in 2007, called the Rotavirus Belgium Impact Study (RotaBIS) [18]. This study showed that there was seasonality in rotavirus infection spread (mostly between January and March); a vaccine herd effect early on; and potentially waning vaccine efficacy to consider when adequately fitting the observed with the modelled data [19,20,21,22]. Moreover, a vaccine catch-up programme to immunise the entire age group up to the age of 5 years was not possible, because the vaccine has a very low frequency of a serious side effect (intussusception) if the doses are not given within strict time schedules [23,24]. Therefore, continuous vaccination of new-born infants with high coverage from the start was needed to obtain control of the infection spread. The follow-up of the observed RotaBIS data identified two key points [22]. First, if the initiation of the vaccination programme was not optimal, this could lead to low vaccine coverage in the group forming the primary source of infection during the normal rotavirus peak season, with the consequence that the herd effect could be low (15%) in the first year and could disappear in the second year due to greater prominence of secondary sources for infection spread [21]. Second, with suboptimal vaccination implementation, the primary source of infection shifted after a while from very young children (less than 13 months old) to an older age group, which may result in long-term regular seasonal peaks of the disease at a lower frequency and height than pre-vaccination. However, the reduced herd effect and the appearance of new smaller disease peaks after a while could be altered with optimal initiation of the vaccination programme, with high coverage from the start (around 90%), and an optimal start date for the vaccination programme (at least 6 months before the next seasonal peak). These findings could be deduced from a more in-depth analysis of the rotavirus vaccination with the RotaBIS follow-up data.

The objective of the present analysis is to evaluate the economic value of an optimal vaccine launch, compared with a non-optimal situation such as the one observed in Belgium. The analysis uses an evaluation technique that allows the simulation of different vaccine launch scenarios, with different long-term accumulated outcome results for the economic assessment. It may identify threshold conditions that determine whether an initial vaccination strategy moves to optimal or less optimal long-term cost-impact results.

## 2. Materials and Methods

Assessing the economic value of rotavirus vaccination in the short to long term, simulating different scenarios, rests on two pillars: the data source and the model.

### 2.1. Data Source

**Figure 1:**
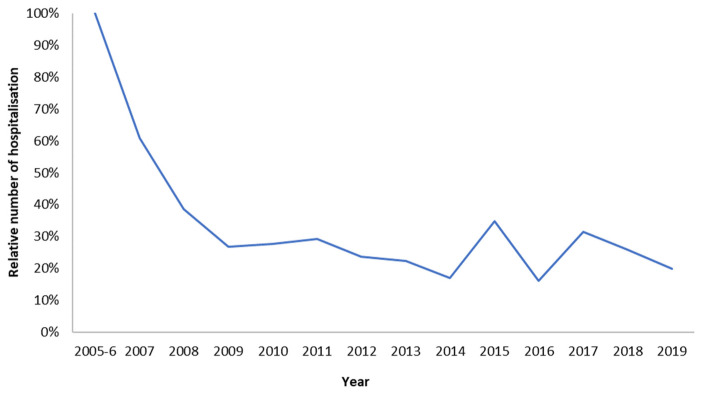
Relative number (%) of rotavirus-specific hospitalisations in years after vaccination introduction (2007–2019) versus pre-vaccination (2005–2006).

Understanding the real-world long-term economic effects of rotavirus vaccination requires the systematic collection of observed data on items causing the high cost of managing the disease and on which the vaccination is known to have a positive impact. Such data were collected in the RotaBIS study, which was initiated in 2007, a year after the vaccine was introduced and partially reimbursed by the Belgian authorities in November 2006 [19,20,25]. Data on disease-specific hospitalisations were retrospectively collected for the years 2005 and 2006, before the introduction of the vaccine. The same information was subsequently gathered annually for 13 years from 11 hospitals, representing the different parts of the country. The following data were assembled for each event, in addition to the test result and date for rotavirus detection: the date of hospitalisation; the specific age when the disease occurred; sex; duration of hospitalisation; and nosocomial acquisition. The full protocol of the study has been reported elsewhere [18]. The information relevant to the present study is summarised in Table 1, showing the numbers of disease-specific hospitalisations by age and year reported over a total period of 15 years (the pre-vaccination years of 2005 and 2006 are reported as average values for the two years combined). Figure 1 presents these observed numbers, showing the reduction in hospitalisations over time after the introduction of the vaccine, with the appearance of new small biennial peaks after 8 years.

### 2.2. The Model

**Figure 2:**
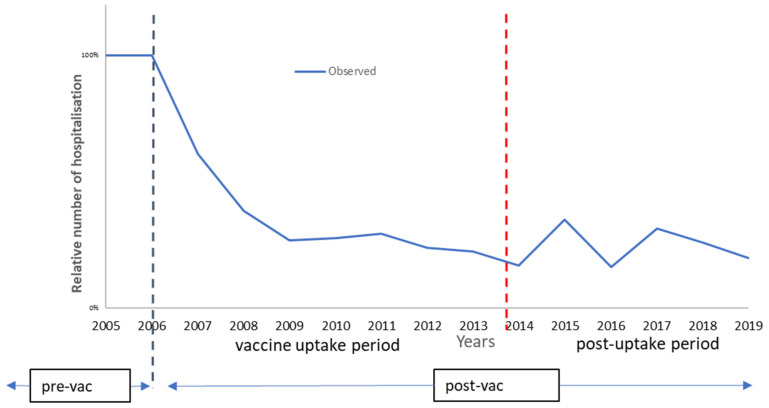
Defining two different periods in the vaccination programme model.

The model needs to replicate the observed data and must include those variables that affect the shape of the observed curve using direct and indirect vaccine effects [22]. The model splits the observation period into two linked consecutive time periods, using a different model structure for each period (Figure 2). Full details of the model, including sensitivity analyses, have recently been presented [22] (see Appendix A and Appendix B for further details on the model input data and model construction).

The first period is the vaccine uptake period that can last 5 to 8 years until a new infection equilibrium has been reached in the target group of children aged ≤5 years. For this period, the model uses a regression equation to characterise the shape of the curve, in which different forces influence the regression line simulating the number of disease-specific hospitalisations observed per year. There are two main forces in the regression equation, each of which combines several components. The first force defines the direct vaccine effects (effectiveness, coverage, and waning). The second force represents the indirect effects of the vaccine (herd effect and secondary sources of infection).

The vaccine uptake period is followed by a post-uptake period, in which the dynamic spread of the infection is simulated using a time differential equation with compartments of susceptible, infectious, and recovered (SIR) groups linked by time-dependent rates of transitions, starting at the hospitalisation level and the time required to replicate the observed biennial disease peaks. The frequency and height of these peaks depend on the entry conditions for the post-uptake period after the vaccine uptake period. These entry conditions include the remaining infection rate in the population, the maintained vaccine coverage rate with its net effect, the susceptible group (new-borns) entering at any given time point, and the contact matrix for the at-risk population (see Appendix B). It is important to note that the initial primary source of infection pre-vaccination shifted in the post-uptake period to an older age group developed during the vaccine uptake period, if the vaccine coverage and the timing of initiating the vaccination were not optimal.

### 2.3. Cost-Impact Analysis

A cost-impact analysis (CIA) was used for this analysis instead of a cost-effectiveness analysis (CEA), because impact evaluation covers the whole vaccinated and unvaccinated at-risk population in which the vaccine has direct and indirect effects, and which is compared with a situation prior to the initiation of the vaccination programme (*pv*) [26]. The calculation for CIA is the same as for CEA using the following formula, in which the cost and effect, once the vaccine is introduced, is the sum of the costs and effects for the unvaccinated (*uv*) group and the vaccinated group (*v*):

$$\mathrm{CIA}=\frac{\Delta C}{\Delta E}$$

$$\Delta C=(C_{pv}-\left(C_{uv}+C_{v}\right))$$

$$\Delta E=(E_{pv}-\left(E_{uv}+E_{v}\right))$$

Δ = difference; *C* = cost; *E* = health effect often expressed in quality-adjusted life-years (QALYs); *pv* = pre-vaccination; *uv* = unvaccinated; *v* = vaccinated

This contrasts with CEA, which evaluates the initially intended vaccinated population, considering the effectiveness of the direct and indirect positive effect of the vaccine such as the herd effect in the control group from within the vaccinated population (the test-negative controls) [27]. Here, the negative indirect effect of the vaccination in the whole population was identified and added in the evaluation when new primary sources of infection in older age groups were created when the vaccination start was not optimal. It makes reference to the impact assessment, as presented by Germaine Hanquet et al. from an epidemiologic perspective, but now applied from an economic view [28].

### 2.4. The Belgian Data

Two comparative CIAs were reported for the Belgian data: one compared the economic results projected prior to market launch with those obtained from the observation of the vaccine uptake period in the RotaBIS study [20,21]; the second compared the predicted data with the long-term observations from the RotaBIS data [19,22]. It should be noted that the pre-marketing economic assessment was developed using a cohort model [29], whereas the comparisons with observed data have a population structured assessment. To make a fair comparison between these two datasets and their model structures, it was necessary to transfer the cohort design into a population model design over time using a multi-cohort approach (see Appendix A). This can be easily achieved for the vaccine uptake period but is challenging for the post-uptake period. It assumes continuity of the effect in the pre-marketing prediction model over time. Simulating small peaks in the post-uptake period would be difficult in a multi-cohort model because of the restriction imposed by the model construct, following individuals in a cohort and not as members of a population. However, the key question is the difference in the reported cost-impact results between pre-launch estimates and post-launch observations. Is the difference the consequence of a real difference in numbers (hospitalisations), an effect of model design (cohort versus population), or due to other factors?

### 2.5. Simulated Scenario Data

This model mimics the hospitalisation rates during the vaccine uptake period and the post-vaccine uptake period of the RotaBIS study. It was used to simulate selecting a better time to start the vaccination programme (August instead of November in the year before the next seasonal disease peak) and immediately reaching a very high vaccine coverage rate (90% instead of 66%). This scenario leads to a higher herd effect during the first year of the vaccine introduction, making it more difficult for the virus to activate secondary infections that produce the small disease peaks later. This is the optimal vaccination introduction scenario (optimal) that should produce substantially improved results compared with the initial launch in Belgium. An intermediate launch scenario was considered with a launch in October and an initial vaccine coverage rate of 67% to evaluate intermediate effects (intermediate) in contrast to the optimum launch strategy (optimal) or the borderline cost-impact results (Belgium). Launch data from other countries in Europe, Finland, the UK, and Spain, [30,31,32] could help to assess these simulations.

### 2.6. Data Input and Output

The input data used to estimate the cost and QALY-loss impact of hospitalisation are presented in Table 2. The cost data are those used when the vaccine was launched in 2006 when it received its reimbursement price in Belgium, which has not changed since. Discounting is applied for costs but not for the QALY health gain associated with vaccination [33,34]. Input values for critical variables that define the shape of the curve during the vaccine uptake period are presented in Table 3 for the observed Belgian data (Belgium), an improved scenario (intermediate), and an optimal design (optimal). 

The output obtained is the incremental cost–impact ratio (ICIR) achieved using different modelling approaches with different scenarios for vaccine launch, listed in Table 4. The launch data from Finland, the UK, and Spain were integrated into the Belgian model of the observed data to estimate ICIR differences with the different launch scenarios in those countries (Table 4). However, it is difficult to compare economic evaluations between countries for obvious reasons, e.g., the price-setting of the vaccine and the hospital cost may differ between countries, and comparisons should be made with caution.

## 3. Results

### 3.1. The Belgian Uptake Period

**Figure 3:**
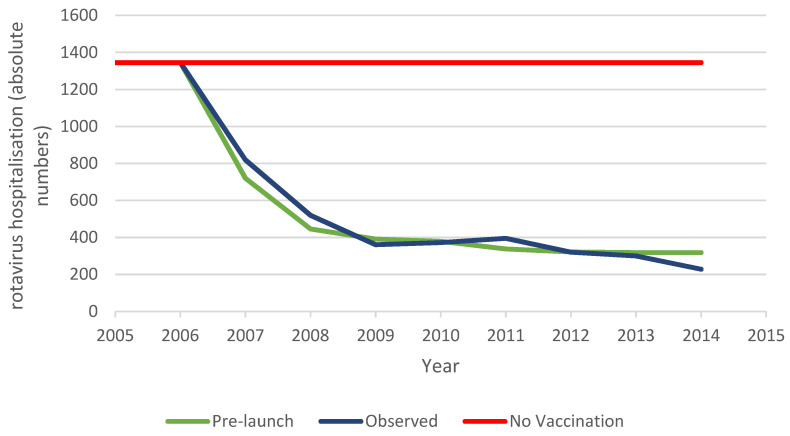
Comparing the hospitalisation reduction data of the pre-launch estimate and the observed data during the uptake period.

The pre-launch predicted hospitalisation reduction, observed hospitalisation reduction data, and predicted hospitalisations with no vaccination are presented for the vaccine uptake period (first 8 years) in Figure 3. The pre-launch prediction for rotavirus vaccination in Belgium is based on the cohort model initially used. For this analysis, a vaccine uptake period was added in a multi-cohort model construction. The model does not capture any herd effect but decreases the vaccine effect over time based on the efficacy trial results from the first versus the second year [1]. The vaccinated birth cohorts are evaluated each year over a 5-year time frame. The accumulated results are compared with the continuous pre-vaccination period where vaccination did not occur (red line in Figure 3). The observed data from the vaccination programme are shown over the same duration of 8 years, but the significant difference from the pre-launch model design is that the full uptake of the vaccination is now included in the data. This shows the effects of the indirect forces of vaccination, related to the herd effect and the presence of secondary sources of infection attenuating the vaccine effectiveness to result in a net effect. 

The cost-impact results are shown in Table 5 for the predicted and observed data, compared with no vaccination.

There is a difference in the ICIR results in favour of the observed data because of a lower use of the vaccine, compared with the pre-launch model. The predicted results reach a similar plateau level in hospitalisations as the observed data, mainly because of the imposed reduction in vaccine effect over time, whereas the plateau level in the observed data is explained by the initial small herd effect, the vaccine coverage rate, and the appearance of secondary sources of infection, while the vaccine effect is maintained at the same level over the period. The observed results therefore broadly achieved the predicted effect on hospitalisations, but at a lower vaccine coverage rate with consequently lower vaccine costs, resulting in a more favourable ICIR than predicted.

### 3.2. The Belgian Long-Term Period

**Figure 4:**
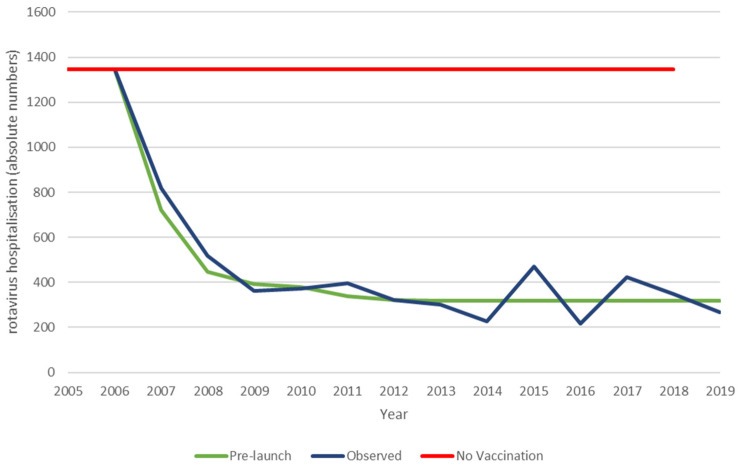
Comparing the hospital reduction data of the pre-launch estimate and the observed data of the whole period.

The next analysis compares the whole period of the observed data with the results from the extension of the pre-launch prediction model, assuming the effect observed after 8 years in the vaccine uptake period is maintained. The observed data differ from the pre-launch prediction by the appearance of small hospitalisation peaks at 9- and 11-years post-vaccine introduction that negatively impact the vaccine effect over time, as shown in Figure 4 and Table 6.

These results now show that the ICIR is more favourable in the predicted data, in contrast with the results for the vaccine uptake period. This difference is explained by the appearance of the hospitalisation peaks in the observed data, causing a marginal gain in the ICIR results for the predicted model. Extrapolation of the observation period beyond 13 years of vaccination with the inclusion of modelled regular biennial peaks over time results in larger changes in the cost-impact results in favour of the extended prediction model. At 18-years post-vaccine introduction, the ICIR results are, respectively, €57,080 for the modelled pre-launch prediction and €65,797 for the observed simulated data, undiscounted (data not shown). The discounted results significantly reduce the difference in the ICIR results (€45,450 and €50,045, respectively).

### 3.3. The Belgian Optimal Evaluation

**Figure 5:**
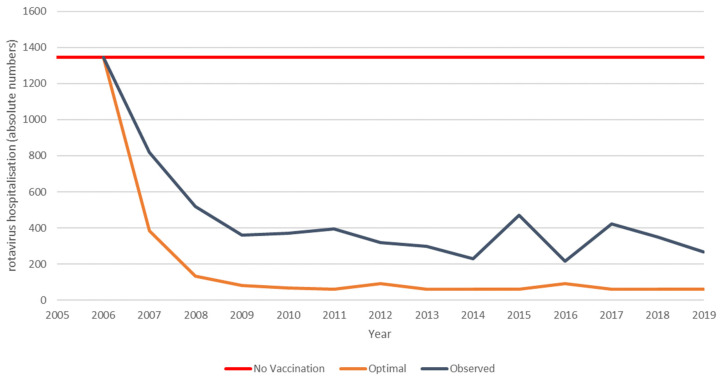
Comparing the hospitalisation reduction in observed data with a simulated optimal introduction over the whole period.

Using the model constructed from the observed data, the vaccination introduction was adjusted by starting vaccination in August instead of November with an immediate coverage rate of 90% instead of 65% (optimal). These adjustments result in a much higher herd effect during the first years of the vaccination programme, which in turn hinders the development of new primary sources of infection in an older age group causing the later disease peaks. Depending on the level of increased herd effect simulated at the start, this scenario produces smaller and less frequent (every 4 years) disease peaks that begin earlier (after 5 years) than those in the observed data (Figure 5). The earlier appearance results from the lower level of infection present in the at-risk population, which means that a new infection equilibrium is reached more quickly. The reduction in hospital events avoided over the period compared with the observed data is impressive (5042 − 1276 = 3766 hospital events or >20% improvement) (Table 7). The high vaccine coverage and consequent vaccine cost mean that the hospitalisation reduction does not produce cost savings compared with no vaccination; however, the total cost is lower compared with the observed data (€30,687,052 − €27,249,792 = €3,437,259 (11% cost gain)).

### 3.4. Scenario Analysis

**Figure 6:**
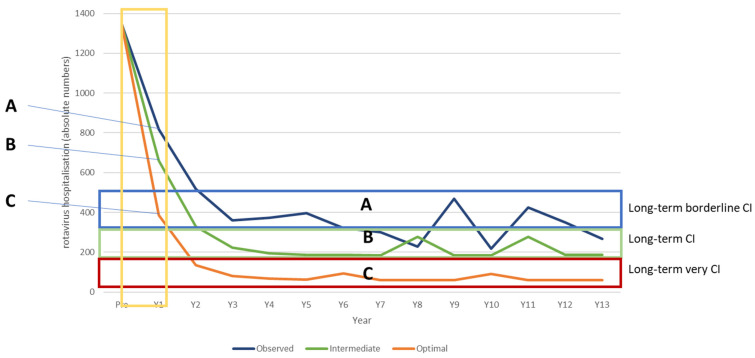
Presenting 3 categories of cost impact (**A**,**B**,**C**) for the rotavirus vaccination long-term effect conditional on its introduction.

The simulation is further used to identify thresholds for vaccination launch parameters that determine when the vaccination produces better cost-impact results overall compared with the observed data. This is illustrated in Figure 6. Three scenarios are modelled, one simulating the observed Belgian data (observed), one using an optimal vaccination introduction (optimal), and one using an intermediate vaccination introduction (intermediate). For each of these scenarios, ranges are applied to key parameters, as specified in Table 8, based on the first-year results of the net effect of the vaccine introduction (indicated by the yellow box in Figure 6). As shown in Figure 6, the long-term hospitalisation reduction level differs considerably between the three scenarios with little overlap, indicated by the blue box (Category A, Belgian observed data); green box (Category B, intermediate vaccination introduction); and red box (Category C, optimal vaccination introduction). The first year determines the category reached in subsequent years because of the correlation between the herd effect in the first year and the appearance of disease peaks due to secondary sources of infection in the subsequent years.

The accumulated reduction in hospitalisations after 13 years of vaccination differs between the categories, reaching at least 65% to 74% (Category A, Belgian observed data); 75–84% (Category B, intermediate vaccination introduction); and >85% (Category C, optimal vaccination introduction). The ICIR is considerably more favourable for the optimal introduction scenario (€10,700) compared with the intermediate introduction scenario (€26,920) and the Belgian situation (€42,356) (Table 8).

### 3.5. Other Countries

**Figure 7:**
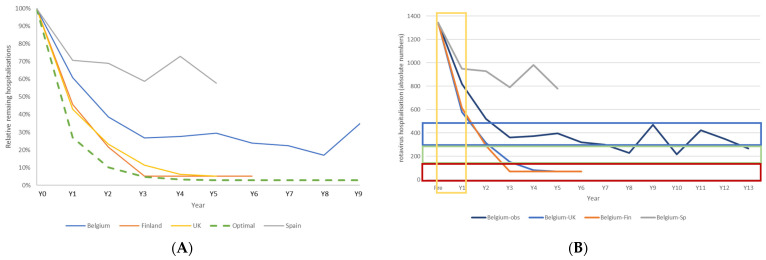
Reporting the annual % of remaining rotavirus hospitalisations for Finland, UK, and Spain (**A**), and reporting the absolute numbers when those % are applied in the Belgian model (**B**), issuing potential cost-impact results in one of the 4 categories of non-ABC, A, B, and C.

Countries across Europe have launched rotavirus vaccination programmes that differ from the Belgian launch in their initial vaccine coverage rates and date of initiating the vaccination programme. Figure 7A shows hospitalisation rates in the vaccine uptake period in three other countries in Europe that introduced rotavirus vaccination and for which data were available in the public domain [30,31,32]. Figure 7B shows the results when these data are applied in the model for the full 13-year period. Finland and the UK introduced the vaccine systematically in 2010 and 2013, respectively, starting earlier in the year than Belgium (September and July, respectively) and with very high immediate vaccine coverage rates. In Spain, the vaccine has been recommended since 2012 but is still not reimbursed, with a consequently wide range of vaccine coverage rates across the country and by year. 

Countries that started vaccination early in the year and with a very high coverage rate from the start (Finland and UK) saw a greater fall in hospitalisations than countries with a less stringent vaccination policy (Belgium and Spain). Finland and the UK appear to be on track to reach the region of the Category C scenario in the model. Long-term vaccination impact is now more difficult to assess because of the effect of preventative measures introduced in response to the COVID-19 pandemic, such as lockdowns, which caused an important additional impact on rotavirus hospitalisations, as observed in Belgium [39]. However, if the modelled predictions of long-term vaccine effect hold, Finland and the UK should expect quite impressive cost-impact results over the long term, which does not appear to be the case for Spain (Figure 7B).

## 4. Discussion

Rotavirus vaccination is an interesting case study to illustrate that there may be potentially important differences in economic value between pre-launch model predictions compared with real-world observational data over time. At the beginning, performing a cost-effectiveness analysis for rotavirus vaccination was considered a straightforward exercise, even with the use of dynamic models, to estimate the potential health gain and price-setting [40,41]. The reality observed in Belgium by the RotaBIS study indicated much greater complexity in infection spread and the vaccine effect. The vaccine launch in Belgium was, by chance, an intriguing case because it was not optimally implemented, but this was not known at the time of reimbursement in November 2006 [42,43,44]. Comparing observed and predicted data made it possible to identify issues in virus spread in the child population, with primary and secondary sources of infections that early rotavirus disease models did not include [11]. Most models assumed a single source of infection that reduced over time with vaccination [45,46]. In addition, the seasonality of the infection implied that there were clear, annual periods of intense virus transmission that should be targeted at the start of the vaccination programme with a very high vaccine coverage of the population transmitting the infection. This was not achieved in Belgium, with the now known consequences [19]. Finally, the vaccination programme did not allow for a catch-up strategy, such as vaccinating a whole age group up to 3 years old at the beginning, because of vaccine safety concerns [23]. This had the consequence of not achieving immediate control of virus spread in children who were older than the target age for vaccination. It was the reason for splitting the evaluation into two periods: a vaccine uptake and a post-vaccine uptake period [22]. All these elements show the importance of a detailed understanding of the pre-vaccination infectious disease situation and patterns of infection spread, before introducing a new vaccine. The vaccine administration process and the potential constraints and safety concerns should be well-known at the start of the vaccination campaign. 

Modelling these elements has helped to clarify the indirect effects of the vaccine that increase or reduce the herd effect, influencing the net vaccine effect and explaining the appearance of new disease peaks over time with a shift to older children as the primary source of infection [22]. The data from Spain confirm the findings in Belgium with a sub-optimal launch of rotavirus vaccination [32,47]. The data from Finland and the UK may prove that initiating the vaccine programme earlier in the year and with an immediately high coverage achieves greater reductions in hospitalisation, compared with what was observed in Belgium [38,48,49]. This suggests that Belgium could have obtained better results by starting the programme differently, although this was not known at the time. After reaching the stage of the post-vaccine uptake period, the modelling results indicate that it would be very difficult to substantially improve the results unless a massive, new intervention shock happened. By chance, such a shock occurred with the lockdowns introduced due to the COVID-19 pandemic in 2020 and 2021, and the rotavirus peaks during those years disappeared in Belgium [39]. This striking result would have been very difficult to achieve without the lockdowns, as increased vaccine coverage does not immediately reduce the primary infection source that shifts to an older age group not directly under the effect of the vaccine. Only the very young ages are vaccinated. This is critical information because when the vaccine programme is not well initiated, it has long-term negative consequences that are difficult to adjust. It is also the situation of the rotavirus vaccination results currently observed in the US [50,51].

A few additional questions could be asked in relation to this economic evaluation. One is about the economic value this vaccine should have pre-launch that defines its price of reimbursement at launch, with a better understanding of the importance of how the vaccine programme is introduced. Analyses relying on simpler models, without taking into account the new knowledge of the optimal method of introducing the vaccination programme, as was carried out in Belgium, may produce a range of cost-effectiveness results in the sensitivity analysis that includes the optimal result. However, such an analysis would not be able to indicate how to achieve the optimal result if not all the necessary details were included in the model construct. In this case, the absence of information on an optimal vaccine introduction to define the price-setting at launch is a risk for both the producer and the paying party. Either may find that they are paying or being paid too much or too little for a vaccine, and it is difficult to readjust the vaccination programme after a non-optimal introduction because of the limitations of readjustment interventions, such as increasing the vaccine coverage rate. Therefore, it is very important to refine the vaccination programme at its introduction to maximise the efficiency of the programme in the long term. Thus, the recommendation is that an economic submission for reimbursement should evaluate different scenarios of vaccine introduction that consider the differences in cost-effectiveness and cost-impact analyses related to the vaccine coverage rate, and the time selected for vaccine introduction, in relation to the expected seasonal disease peak. This approach, with an emphasis on obtaining initial high coverage ahead of the next expected seasonal peak, could be applicable to other diseases with marked seasonality and high contagion. If COVID-19 becomes an endemic disease in infants with seasonal peaks, these findings may be relevant to future research on the design of a potential COVID-19 vaccination programme in this age group. Could this have been foreseen in the Belgium submission file for the rotavirus vaccine? This would have been difficult if the full infection spread was not well understood at the beginning, having identified the importance of an optimal introduction of the vaccination and having discovered the age shift in the primary source of infection after a sub-optimal introduction. In this respect, one should remember that the European randomised controlled trial (RCT) conducted for Rotarix in 2004–2006 had a randomisation process of two vaccinated children for one placebo child [1]. This type of randomisation increased the herd effect in the placebo group, as the randomisation occurred at local level and not by a cluster site. In cluster site randomisation, regions are divided into clusters. The clusters are randomised to vaccination or no vaccination, thereby reducing the chance of a herd effect occurring in the unvaccinated clusters. In contrast, in local randomisation, the control group is subject to herd effects, resulting in an apparently decreased vaccine effect in the second year of the evaluation. This was not considered when the analysis was conducted and reported because of the lack of baseline information prior to the vaccine introduction. It is also possible that other, additional factors may influence the observed local results, such as the organisation of day-care centres and their potential function as a hub of local epidemics, which would not have good infection control and have poor vaccine coverage. However, there are limitations on the complexity of models that can be constructed and applied in practice. Factors that do not have critical effects on vaccine impact or do not cause important costs or health changes may add little to the economic value generated by the more complex model. The right balance needs to be found between the feasibility of collecting and analysing sufficient data and the wants and needs of the paying parties and producers. The precise balance is likely to vary between specific interventions and settings.

Finally, is this economic model also applicable to other settings such as non-high-income countries? Some critical points that are essential for the optimal functioning of the vaccine in high-income countries but that could be absent in other countries include the seasonality of infection spread, easy contact patterns among very young children (such as day-care centres) that facilitate virus transmission, and the hospitalisation of severe cases leading to a high healthcare cost. It would be a challenge to apply the current model if any of those conditions were not fulfilled. Nevertheless, this analysis indicates that rapidly achieving high coverage at the start of a rotavirus vaccination programme is essential for maximising the health benefit of the vaccine, as this minimises the development of secondary sources of infection that persist over time and are very difficult to correct at a later stage.

The analysis presented here has some limitations. Some cost items, such as first-line support and indirect costs, were not considered, and not all the QALY losses at different disease stages were included in this evaluation. However, the focus of the analysis was to demonstrate that quite different economic value results could be obtained for a vaccine from a predicted pre-launch value assessment and real-life data observations. Vaccination needs data monitoring on its effect over time once approved and implemented, in order to capture deviations from what could be considered an optimal launch. The economic analysis is an additional tool to help in the selection of a vaccine strategy. For instance, some countries like to produce price–volume contracts when introducing new vaccines. These results suggest that such a policy would be a disaster for rotavirus vaccination if the volume is fixed at 40% or 50% for the first year of implementation followed by progressive increases in vaccine coverage over time. With a start at 40–50% vaccine coverage across a country, the present model suggests that it is likely that a limited effect will be seen on hospitalisation reduction, limiting the total value of the vaccine in the short to long term. Conversely, this model suggests that obtaining immediate very high vaccine coverage ahead of the next expected seasonal disease peak would maximise both the health benefit of rotavirus vaccination and its cost impact over the long term.

## 5. Conclusions

Pre-launch economic assessments of new vaccines against rotavirus in high-income countries should be considered very carefully, as modelling based on observational data from Belgium indicates that the long-term vaccine benefit strongly depends on the details of the vaccination introduction. Issues such as changes in the infection spread and the consequent effects on vaccine impact make rotavirus vaccination assessment much more complex than initially thought, with quite disparate economic results depending on whether the initial vaccine introduction was optimal or sub-optimal.

## Figures and Tables

**Table 1:** Number of rotavirus hospitalisations by age and year (m—month; Y_n_—year number).

Age/Yn	2005–2006	2007	2008	2009	2010	2011	2012	2013	2014	2015	2016	2017	2018	2019
0–2 m	113	94	62	56	44	65	54	44	48	56	28	55	52	27
3–12 m	678	340	152	129	127	133	103	97	70	137	75	123	125	95
13–24 m	413	311	208	100	139	134	114	107	74	186	85	180	119	96
25–36 m	102	56	67	49	33	44	33	33	31	67	17	42	37	35
37–48 m	27	16	18	19	19	12	9	15	4	13	8	18	9	9
49–60 m	12	2	12	8	10	7	7	4	1	10	4	6	8	6
Total	1345	819	519	361	372	395	320	300	228	469	217	424	350	268

**Table 2:** Cost and QALY loss input data used to measure the cost impact of rotavirus vaccination over time.

Variable (Name)	Unit Value	Number	Total	Reference
Hospitalisation Pre-vaccination cost	€1467	7 days	€10,269	[35]
Hospitalisation Post-vaccination cost	€1467	5 days	€7335	[25]
Vaccine cost (Rotarix)	€70/dose	2	€140/vaccination	[34]
QALY-loss Pre	−0.47/hospital day	7 days	−0.009	[36]
QALY-loss Post	−0.47/hospital day	5 days	−0.006	[25]
Target population to vaccinate pre-vaccination	5%	791	15,820	[19]
Discounting cost	3%			[37]

**Table 3:** Critical input data values for the uptake period of Belgium, intermediate scenario and optimal scenario.

Variable (Name)	Code	Belgium	Intermediate	Optimal
Vaccine efficacy	VE	0.95	0.95	0.95
Vaccine coverage focused	VCF	0.66	0.67	0.88
Vaccine coverage routine	VCR	0.86	0.87	0.95
Herd effect non-indicated	Hn	0.41	0.42	0.68
Secondary infection source herd	SI_h_	0.10	0.06	0.01
Start month vaccination	Sm	Nov	Oct	Aug

Focused: during the first months of vaccination prior to reaching the routine coverage; routine: reaching the normal coverage of child vaccination; herd effect non-indicated: herd effect amongst those who could not receive the vaccine.

**Table 4:** Different scenarios for calculated ICIR, in which the comparator is no vaccination.

Scenario Number	Scenario	Cases	Period	Evaluation	Reference
1	Belgium prediction pre-launch	Estimated with multi-cohort model	8 years		[29]
2	Vaccine uptake period	Observed data (RotaBIS)	8 years	1 with 2	[20,21]
3	Belgian prediction pre-launch	Estimated with multi-cohort model	15 years		
4	Vaccine uptake and post-uptake period	Observed data (RotaBIS)	15 years	3 with 4	[19]
5	Vaccine uptake and post-uptake period	Optimal simulation from RotaBIS data	15 years	4 with 5	[22]
6	Finland (2009)	Observed data	6 years	4 with 6	[30,38]
7	UK (2013)	Observed data	5 years	4 with 7	[31]
8	Spain (2013)	Observed data	5 years	4 with 8	[32]

**Table 5:** Cost-impact results comparing the days of hospitalisation regarding no-vaccination, pre-launch predicted data, and vaccinated observed data for the vaccine uptake period.

Item	Undiscounted				Discounted			
	Age Group	No Vaccination	Pre-Launch Predicted	Vaccinated Observed	Age Group	No Vaccination	Pre-Launch Predicted	Vaccinated Observed
Hospital days	0–2 m	904	168	467	0–2 m	904	168	467
	3–12 m	5424	1010	1151	3–12 m	5424	1010	1151
	13–24 m	3304	1339	1187	13–24 m	3304	1339	1187
	25–36 m	816	482	346	25–36 m	816	482	346
	37–48 m	216	155	112	37–48 m	216	155	112
	49–60 m	96	76	51	49–60 m	96	76	51
	Total	10,760	3231	3314	Total	10,760	3231	3314
Cost	Hospital cost	€15,784,920	€3,385,608	€3,472,599	Hospital cost	€14,266,180	€3,112,588	€3,213,999
	Vaccine cost		€15,503,600	€14,219,016	Vaccine cost		€13,988,287	€12,769,962
QALY	QALY-loss	−96.99	−20.80	−21.34	QALY-loss	−96.99	−20.80	−21.34
	**ICIR**		**€40,747**	**€25,204**	**ICIR**		**€37,208**	**€22,707**

m: month; QALY: Quality Adjusted Life year; ICIR: Incremental Cost Impact Ratio; €: Euro.

**Table 6:** Cost-impact results comparing the days of hospitalisation regarding no vaccination, pre-launch predicted data, and the vaccinated observed data of the whole period.

Item	Undiscounted				Discounted			
	Age Group	No Vaccination	Pre-Launch Predicted	Vaccinated Observed	Age Group	No Vaccination	Pre-Launch Predicted	Vaccinated Observed
Hospital days	0–2 m	1469	258	685	0–2 m	1469	258	685
	3–12 m	8814	1567	1706	3–12 m	8814	1567	1706
	13–24 m	5369	1954	1853	13–24 m	5369	1954	1853
	25–36 m	1326	703	544	25–36 m	1326	703	544
	37–48 m	351	229	169	37–48 m	351	229	169
	49–60 m	156	112	85	49–60 m	156	112	85
	Total	17,485	4823	5042	Total	17,485	4823	5042
Cost	Hospital cost	€25,650,495	€5,054,124	€5,283,296	Hospital cost	€21,613,510	€4,353,844	€4,568,730
	Vaccine cost		€25,470,200	€25,403,756	Vaccine cost		€21,410,855	€21,161,515
QALY	QALY-loss	−157.60	−31.05	−32.46	QALY-loss	−157.60	−31.05	−32.46
	**ICIR**		**€38,513**	**€40,247**	**ICIR**		**€32,803**	**€32,896**

m: month; QALY: Quality Adjusted Life year; ICIR: Incremental Cost Impact Ratio; €: Euro.

**Table 7:** Cost-impact results of comparing the overall observation period between no vaccination, vaccinated observed data, and simulated vaccinated optimal introduction.

Item	Undiscounted				Discounted			
	Age Group	No Vaccination	Vaccinated Observed	Vaccinated Optimal	Age Group	No Vaccination	Vaccinated Observed	Vaccinated Optimal
Hospital days	0–2 m	1469	685	351	0–2 m	1469	685	351
	3–12 m	8814	1706	281	3–12 m	8814	1706	281
	13–24 m	5369	1853	431	13–24 m	5369	1853	431
	25–36 m	1326	544	144	25–36 m	1326	544	144
	37–48 m	351	169	44	37–48 m	351	169	44
	49–60 m	156	85	25	49–60 m	156	85	25
	Total	17,485	5042	1276	Total	17,485	5042	1276
Cost	Hospital cost	€25,650,495	€5,283,296	€1,336,632	Hospital cost	€21,613,510	€4,568,730	€1,190,656
	Vaccine cost		€25,403,756	€25,913,160	Vaccine cost		€21,161,515	€21,410,855
	Cost difference (Ob-Op)			€3,437,259				€3,128,733
QALY	QALY-loss	−157.60	−32.46	−8.21	QALY-loss	−157.60	−32.46	−8.21
ICER	ICIR (NV-Op)		€40,247	€10,705	ICIR (NV-Op)		€32,896	€6,613
	ICIR (Ob-Op)			−€141,746	ICIR (Ob-Op)			−€129,023

m: month; QALY: Quality Adjusted Life year; ICIR: Incremental Cost Impact Ratio; €: Euro; NV: no vaccination; Op: optimal; Ob: observed).

**Table 8:** Threshold value ranges of critical parameters in first and subsequent years in category A, B, and C with summary results per category.

**Variable Input Values**			**Category A**			**Category B**			**Category C**	
**Time**	**Type of Variable**	**Code**	**Base Case**	**Min**	**Max**	**Base Case**	**Min**	**Max**	**Base Case**	**Min**
First year	Vaccine coverage	VCF	0.66	0.49	0.75	0.67	0.38	0.73	0.88	0.74
	Herd effect	Hn	0.41	0.20	0.52	0.42	0.06	0.50	0.68	0.50
Subsequent years	Vaccine coverage	VCR	0.86	0.81	0.87	0.87	0.85	0.89	0.95	0.89
	Secondary infection	Siv	0.10	0.08	0.05	0.06	0.09	0.05	0.01	0.03
**Results**			**Category A**			**Category B**			**Category C**	
Remaining hospital events			5042	6120	4546	3259	4371	2798	1275	2623
% reduction			71%	65%	74%	81%	75%	84%	93%	85%
Incremental cost–impact ratio (ICIR) (undiscounted)			€42,356	€56,403	€39,216	€26,920	€37,424	€22,838	€10,700	€21,400

Min: minimum value; Max: maximum value; ICIR: incremental cost impact ratio; No maximum values are presented for Category C because, with the high baseline values of VE (see Table 3), VCF, and VCR, it is difficult to further increase these values in reality.

**Figure A1:**
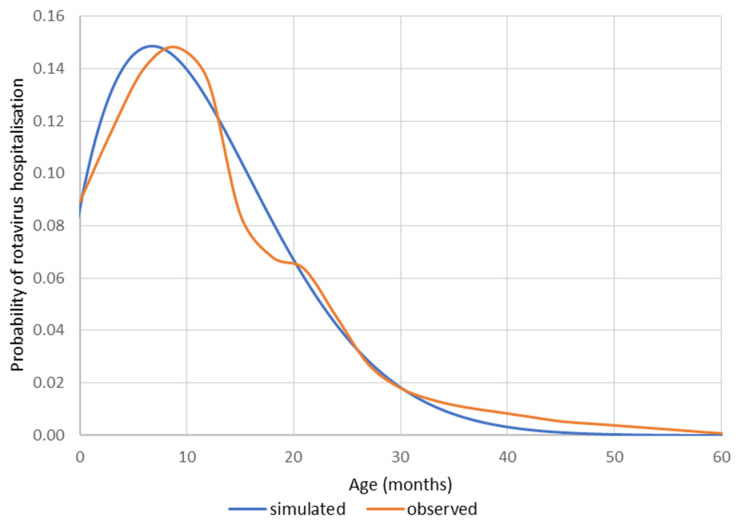
Distribution of rotavirus hospitalisations as a function of age (months).

**Figure A2:**
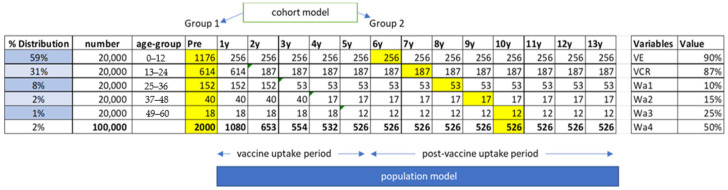
The difference in cohort modelling and population modelling structure applied in rotavirus vaccination assessment with the variable assessment. m: month; VE: Vaccine Efficacy; VCR: Vaccine Coverage Rate; Wa: Waning rate (1 after 1 year; 2 after 2 years; 3 after 3 years; 4 after 4 years).

**Figure A3:**
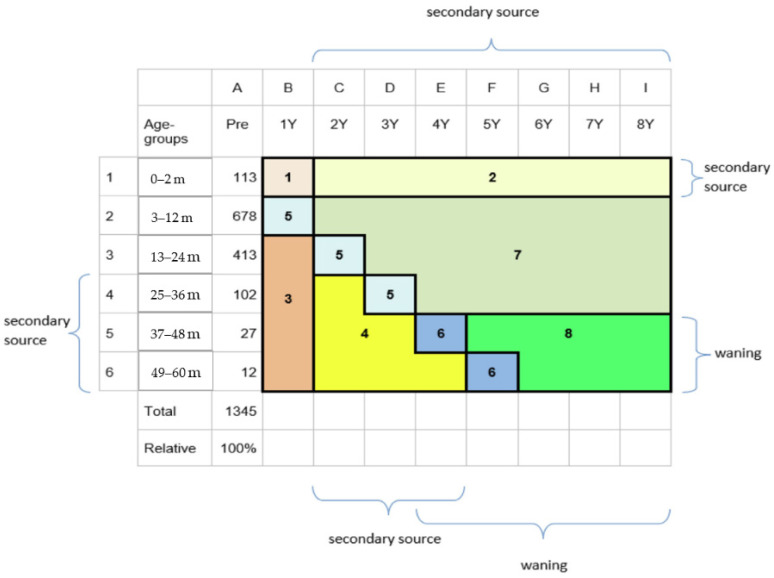
Identifying the different effects by age group and time point for the vaccine direct and indirect effects during the vaccine uptake period. m: month; Y: year.

**Figure A4:**
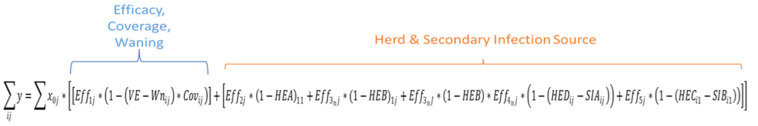
The applied regression equation and input data used to replicate observed hospitalisations during the vaccine uptake period. i=year*,* j=age−group*,* y=rotavirus hospitalisations post−vaccination*,* $x_{0j}=rotavirus\;hospitalisations\;pre-vaccination$. *Other codes are explained in* *Table A1*.

## Data Availability

This analysis only used published information. The models and results presented in this paper can be obtained from the author on request.

## Appendix A. Transforming the Cohort Model Design into a Population Model Structure

The initial economic assessment of rotavirus vaccines, published around the period of approval and launch in Europe in 2006, used a static Markov cohort model as a reference, published by Melliez et al. [52] and applied to Belgium [29] and many other countries in Europe (including the UK [40], France [2], Italy [53], the Netherlands [54], and Germany [55]). The evaluation was performed as if the vaccine was assessed after a period of vaccine uptake where the at-risk population (children ≤ 5 years old) was followed from birth until the age of 5 years (the at-risk period). Pre-vaccination, the rotavirus hospitalisation rate in high-income countries was known to be concentrated in the age group up to 2 years old, following a Weibull distribution (Figure A1).

The effect of the vaccine in the vaccinated cohort was predicted and modelled by only three variables: vaccine effect (VE), vaccine coverage (VRC), and vaccine waning (Wa). The last element was heavily promoted by academic research groups based on the reduced effect of the vaccine reported in the RCT when comparing the first and second year [1]. There was, however, no evidence that vaccine waning was really occurring very early on after its introduction. After 5–6 years of following the vaccine effect annually using a standard approach of registering disease-specific hospitalisations, it was seen that the hospitalisations were reaching a plateau over time after 3 years of vaccination (RotaBIS study) [20,21]. The level of plateau formation could not be explained by a process of vaccine waning, as suggested by early assessments on the vaccine effect. Waning should happen very early and with a dramatically high level that did not match the model constructed to simulate the observed data.

Other effects could better explain the plateau formation than vaccine waning. These include other forces of the vaccine effect such as the herd effect and the activation of secondary or other sources of infection than the primary one, not covered by the vaccination. To illustrate the presence of those other forces causing the plateau formation, a dataset that demonstrated those effects was needed, and it became clear that a population model instead of a cohort approach was the right design to illustrate those new effects [56].

Since the data assembled in the RotaBIS study could be easily presented as a population-structured design (a cross-sectional annual evaluation of the at-risk population subdivided into different age groups), the initial cohort model design needed to be transformed into a population design to be capable of making a fair comparison between predicted and observed results.

Figure A2 uses a hypothetical example to show how this model was constructed. In the initial cohort design, only the cells in yellow were assessed and included in the model. Group 1 was the no-vaccination group and Group 2 was the vaccinated cohort group in which the level of vaccine waning was adjusted in each age group, while the vaccine effect and coverage remained the same. For the population model approach, the full length of the model structure was used from Year 1 (Y1) until the last year of observation in the RotaBIS study (Y13). Therefore, it included the vaccine uptake period as a necessary condition for the comparison between the prediction approach and the observed data.

## Appendix B. Developing the Model Structure to Assess the Disease Burden and Vaccine Impact in the Short to Long Term

Capturing the indirect effect of a vaccine in a model, causing what is called the herd effect (protecting unvaccinated individuals by a lower circulation of the infectious agent due to vaccination), can be easily demonstrated with the development of a compartmental dynamic model, in which a critical element obtains data about the contact matrix by which the virus transmission can be quantified over time. This approach was applied after the first cohort models were published, with the additional sophistication that individuals could be reinfected over time, with a better immune response the more times they had been infected [45,46,57,58].

Including the herd effect (Hn) in the model was important, but this additional element, besides vaccine effect (VE), coverage (VCR), and waning (Wa), was not sufficient to fully explain the observed effects if the conditions of the vaccine implementation were not optimal, as was the case for Belgium [21]. If the vaccine launch had been optimal in Belgium, it would not have been possible to discover that there was another element playing a critical role in the observed data, which was the presence of secondary sources of rotavirus infection. Those secondary sources manifested themselves indirectly by reducing, in the second year after the introduction of the vaccine, the level of herd effect that would have been expected. This issue of herd effect disappearance in the second year could only be explained if some other source of rotavirus infection was present to contaminate the unvaccinated population that would otherwise have been protected by the herd effect. This was called cannibalisation, as those individuals normally protected by the herd effect were suddenly unprotected and were attacked and infected by a new infection group within the at-risk group that differed from the primary source of rotavirus infection. This phenomenon was temporarily present as long as the potential herd effect across unvaccinated age groups was not covered by the normal vaccine procedure. It took 5 years in total for the whole population to be covered by vaccination.

The secondary sources of rotavirus infection grew to become an ongoing new source of infection, causing new peaks at a lower frequency and height than pre-vaccination. To incorporate this process of cannibalisation into a dynamic compartmental model is a challenge, as more data and more specific interaction data are required that are not easy to assemble. Moreover, the type of statistical analysis needed to solve the more complex time-differential equation was not straightforward to compute. A simpler solution was therefore followed to model the effects during the vaccine uptake period, using a regression equation in which each of the elements affecting hospitalisations during that period was specified by its effect for each sub-period, as defined in Figure A3 and Figure A4, and Table A1.

**Table A1:** Input values for the simulation fitted to the observed Belgian data (value BE), for an ‘ideal’ or optimal simulation maximising the reduction in hospitalisation (value ideal).

Variable/Force			Uncertainty		Value Ideal	Source
	Code	Value BE	Min	Max		
Vaccine efficacy	**VE**	**95%**		95%	99%	[34]
Vaccine coverage 1st year	**Cov_1j_**	**52%**	49%	54%	85%	[7,24,35]
Vaccine coverage subsequent years	**Cov_ij_**	**83%**	82%	85%	98%	[7,24,35]
Herd effect (0–2 m 1st year)	**HEA**	**15%**	13%	17%	50%	[7]
Herd effect (older unvaccinated 1st year)	**HEB**	**31%**	29%	33%	83%	[7]
Herd effect (older unvaccinated subsequent years)	*HED*	*33%*	30%	45%	87%	Assumption
Herd effect (0–2 m subsequent years)	*HEC*	*75%*	60%	80%	80%	Assumption
Secondary infection source (2nd year older)	*SIA*	*35%*	30%	45%		Assumption
Secondary infection source (0–2 m subsequent years)	*SIB*	*27%*	25%	35%		Assumption
Waning cohort	*Wn*	*12%*	5%	20%		Assumption
Effect variables	*Eff_nj_*	Binary variables (0,1) that activate or disactivate part of the equation				

BE, Belgium; m, month. Bold text indicates parameter values directly obtained from the RotaBIS data. Italic text indicates best estimates taking a conservative approach.

The model grid structure is split into eight different areas. Area 1: no vaccination, limited herd effect of the first year vaccine coverage; area 2: no vaccination, herd effect under the vaccine coverage of subsequent years; area 3: no vaccination, herd effect under vaccine coverage first year; area 4: no vaccination, herd effect and secondary sources of infection appearing; area 5: vaccination first year with vaccine effect in the cohort over two additional years; area 6: vaccination first year with vaccine effect and vaccine waning starting in year four post-vaccine introduction; area 7: vaccination under vaccine coverage of subsequent years; area 8: vaccination at vaccine coverage of subsequent years with vaccine waning.

Once a new infection equilibrium was reached with vaccine effect and coverage forming a regular pattern, it was then possible to apply a more straightforward dynamic, time-differential, compartmental model (susceptible, infectious, recovered). This was conditional on the presence of the infection level reached at the end of the vaccine uptake period, which was applied at the start of the post-vaccine uptake period developed in the model construct [22]. The model output of the post-vaccine uptake period indicated that the frequency and height of the long-term disease peaks depend on the level of infection present in the group.

The present manuscript adds the new finding that the appearance of the infection equilibrium level may develop sooner with a lower infection level at equilibrium. There are reasons to believe that if less virus is circulating, the infection equilibrium is more easily reached and may be reached more rapidly. This hypothesis needs to be confirmed by real observed data. However, ultimately, the economic end-result may not be strongly affected by whether those very small new peaks appear sooner or later.
